# Development of Stereocomplex Polylactide Nanocomposites as an Advanced Class of Biomaterials—A Review

**DOI:** 10.3390/polym15122730

**Published:** 2023-06-19

**Authors:** Muhammad Samsuri, Purba Purnama

**Affiliations:** 1 Chemical Engineering Department, Universitas Bhayangkara Jakarta Raya, Bekasi 17121, West Java, Indonesia; msamsuri1979@ubharajaya.ac.id; 2 School of Applied STEM, Universitas Prasetiya Mulya, Tangerang 15339, Banten, Indonesia; 3 Vanadia Utama Science and Technology, PT Vanadia Utama, Jakarta 14470, Indonesia

**Keywords:** polylactide, stereocomplex, nanoparticles, biodegradable polymers, advanced materials, interfacial interaction, self-assembly, nucleating agent, thermal and mechanical properties

## Abstract

This review paper analyzes the development of advanced class polylactide (PLA) materials through a combination of stereocomplexation and nanocomposites approaches. The similarities in these approaches provide the opportunity to generate an advanced stereocomplex PLA nanocomposite (stereo-nano PLA) material with various beneficial properties. As a potential “green” polymer with tunable characteristics (e.g., modifiable molecular structure and organic–inorganic miscibility), stereo-nano PLA could be used for various advanced applications. The molecular structure modification of PLA homopolymers and nanoparticles in stereo-nano PLA materials enables us to encounter stereocomplexation and nanocomposites constraints. The hydrogen bonding of D- and L-lactide fragments aids in the formation of stereococomplex crystallites, while the hetero-nucleation capabilities of nanofillers result in a synergism that improves the physical, thermal, and mechanical properties of materials, including stereocomplex memory (melt stability) and nanoparticle dispersion. The special properties of selected nanoparticles also allow the production of stereo-nano PLA materials with distinctive characteristics, such as electrical conductivity, anti-inflammatory, and anti-bacterial properties. The D- and L-lactide chains in PLA copolymers provide self-assembly capabilities to form stable nanocarrier micelles for encapsulating nanoparticles. This development of advanced stereo-nano PLA with biodegradability, biocompatibility, and tunability properties shows potential for use in wider and advanced applications as a high-performance material, in engineering field, electronic, medical device, biomedical, diagnosis, and therapeutic applications.

## 1. Introduction

Polylactide (PLA) is a thermoplastic and aliphatic polyester that is widely used in various applications. PLA can be synthesized via the polymerization of lactide, a cyclic dimer produced by lactic acid’s dehydration process. Poly(L-lactide) (PLLA), poly(D-lactide) (PDLA) and poly(D,L-lactide) (PDLLA) are synthesized through the ring-opening polymerization of three stereoisomers of lactide: L-lactide, D-lactide and meso-lactide (D,L-lactide), respectively. PLA is known as “green” polymers for replacing fossil-based polymers because it is synthesized from renewable resources with biodegradability characteristics. PLA is now being used in the packaging industry as a substitute for fossil-based polymers such as polyethylene terephthalate (PET), polyvinyl chloride (PVC), and high-impact polystyrene (HIPS). For wider application in high-performance materials, PLA has drawbacks in terms of its mechanical and thermal properties (e.g., heat deflection temperature). Because of its limitations compared to traditional polymers, PLA should be treated to improve its properties [1]. There are various approaches to enhance the properties of PLA, for instance, stereocomplexation and nanocomposites.

In the previous decades, the development of PLA material through stereocomplexation and nanocomposites have attracted considerable interest in both academia and industry. The formation of stereocomplex PLA (s-PLA) was discovered in the enantiomeric PLLA and PDLA blends because of the strong interaction between the L-lactide and D-lactide fragments [2]. The D- and L-lactide segments of enantiomeric PLA homopolymers were proposed to form CH_3_–O=C interactions [3,4]. These interactions caused PLLA and PDLA chains to be packed side by side as s-PLA crystallites [5], which enhanced the mechanical and thermal properties of the materials [2,6,7,8,9,10,11,12,13,14].

Polymer nanocomposites have been developed as a combination of organic polymer materials and nanoscale inorganic nanoparticles (<100 nm) [15,16,17]. In PLA-based material development, the various dimensionalities of the dispersed nanosized filler in the PLA matrix influence the enhancement of mechanical, physical, thermal, optical, hydrolytic and thermal degradation, and other properties of PLA homopolymers [2,4,17,18,19]. The s-PLA and PLA nanocomposites show similarities regarding the enhancement of their properties and synthesis routes (e.g., solution casting, melt blending, irradiation, and supercritical fluid technology [20,21,22,23,24,25,26,27].

The degradability, sustainability, and tunability of materials are some of the most important challenges in current and future material development. Because of its biodegradability and sustainability, PLA is a promising material for replacing non-degradable materials. By combining stereocomplexation and nanocomposite approaches to generate stereo-nano PLA, the synergistic effect of both approaches is expected to enhance the properties of the materials and create the opportunity for its use in advanced performance applications [19,28,29,30]. The development of stereo-nano PLA enables the design and modification of biodegradables materials so that an eco-friendly product with high-performance, specially designed, and engineered properties is achieved.

Some studies have been conducted on s-PLA and PLA nanocomposites [11,13,31,32,33,34]. In the s-PLA field, some reports focused on synthesis and the formation of s-PLA crystallites with various variables (e.g., molecular weight, concentration, and processing methods), property improvements, and some applications of s-PLA based materials [11,13,31]. On the other hand, reports of PLA nanocomposites focused on the nanocomposite developments and property enhancements as consequences of the incorporation of nanoparticles in the PLA matrix, followed by some applications [32,33,34]. Recently, a report provided a systematical study focused on a combination of PLA-based polymers and specific organic nano filler cellulose-based nanoparticles [32].

Although some reports on the development of s-PLA and PLA nanocomposites are available, the aim of this article is to present the most important development in stereo-nano PLA. In this review, we highlight the challenges and recent development of stereo-nano PLA, including the synthesis, properties, and prospective applications of this material. Some general aspects of s-PLA and PLA nanocomposites are discussed first. We believe that this review will provide meaningful guidance on the recent development and future potential of stereo-nano PLA materials.

## 2. Stereocomplex Polylactide (s-PLA) and Polylactide (PLA) Nanocomposites

### 2.1. Stereocomplex PLA (s-PLA) Synthesis and Properties

**Figure 1:**
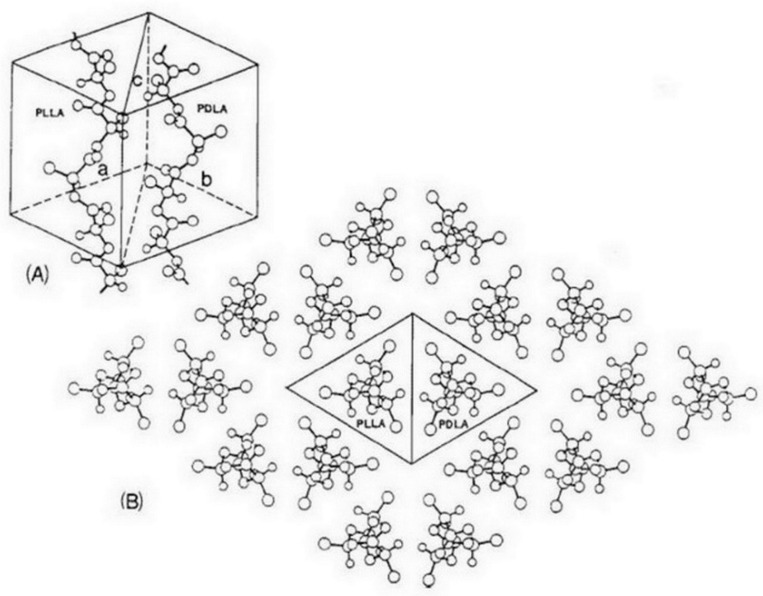
Crystal structure of s-PLA. (**A**) Structural model of s-PLA crystal from PLLA and PDLA. (**B**) Molecular arrangement projected on the plan normal to the chain axis. Reproduced from [5], with permission from Taylor & Francis, 2022.

In the last three decades, stereocomplex crystalline formation (stereocomplexation) and the addition of nanosized fillers (nanocomposites) have been used to enhance the properties of PLA materials. The stereocomplexation of PLA was first reported by Ikada and coworkers in 1987, in which a complex material (later called s-PLA) was obtained from an equimolar solution of PLLA and PDLA homopolymers [2]. The presence of L-lactic acid and D-lactic acid chains resulted in a strong interaction to form a s-PLA crystalline lattice (Figure 1) [5]. The hydrogen bonding between CH_3_–O=C of PDLA and PLLA chains was the driving force behind the formation of new crystalline materials which enhanced the properties of PLA homopolymers [6]. s-PLA has a different crystal structure and a higher melting temperature (by approximately 50 °C) than optically pure PLA homopolymers have. This is caused by the three-dimensional (3D) interaction involved in intermolecular hydrogen bonding [3,4,5]. The strong interaction between enantiomer lactide fragments also enhances the thermal and mechanical properties of PLA, including its degradation resistance [6,7,8,9,10,11,12,13].

Generally, the s-PLA can be obtained from the mixture of PLA homopolymers in the presence of a solvent, melt state, or supercritical fluid (Table 1). Because of s-PLA has a different crystal structure, its presence can be confirmed via DSC and wide-angle X-ray spectroscopy (WAXS) analysis [2]. s-PLA has a higher melting temperature (by approximately 50 °C) compared to its homopolymers. In WAXS profiles, the main peaks of s-PLA (*X_D_* = 0.5) are observed at 2*θ* values of 12°, 21°, and 24° and 19°, whereas the PLA homopolymer peaks (*X_D_* = 1) appeared at 2*θ* values of 15° and 17° [2]. The formation of crystalline s-PLA depends on the molecular weight of homopolymers [14]. Well-mixed PLA homopolymers blend with flexible L- and D-lactide chain mobility affects to the lower critical concentration of s-PLA crystallites. PLA homopolymers with a low molecular weight will simply dissolve in an organic solvent and at a low melting temperature. The solubility of a PLA homopolymer in an organic solution and its melt state at the melting point affects the crystal formation of the materials. In a mixture of enantiomers (solution or melt state), there is a competition for the formation of s-PLA and the crystalline homopolymer. s-PLA can be easily obtained from low-molecular-weight PLLA/PDLA blends because the high-molecular-weight PLA homopolymer has limited-mobility L- and D-lactide chains. The PLLA: PDLA ratio also affects the formation of s-PLA crystallites, with a ratio of 1:1 being the optimum composition [4,14,16,18]. The optical purity of the PLA homopolymers also determined the formation of crystalline s-PLA because the interaction between the L- and D-lactide chains is responsible for it [16,17]. The combination of the PLA homopolymer ratio, molecular weight, concentration, solvent selection (in solution-based methods), and temperature (in melting process) affects the processing time to produce s-PLA crystallites [19,20,21,22,23,24,25].

**Figure 2:**
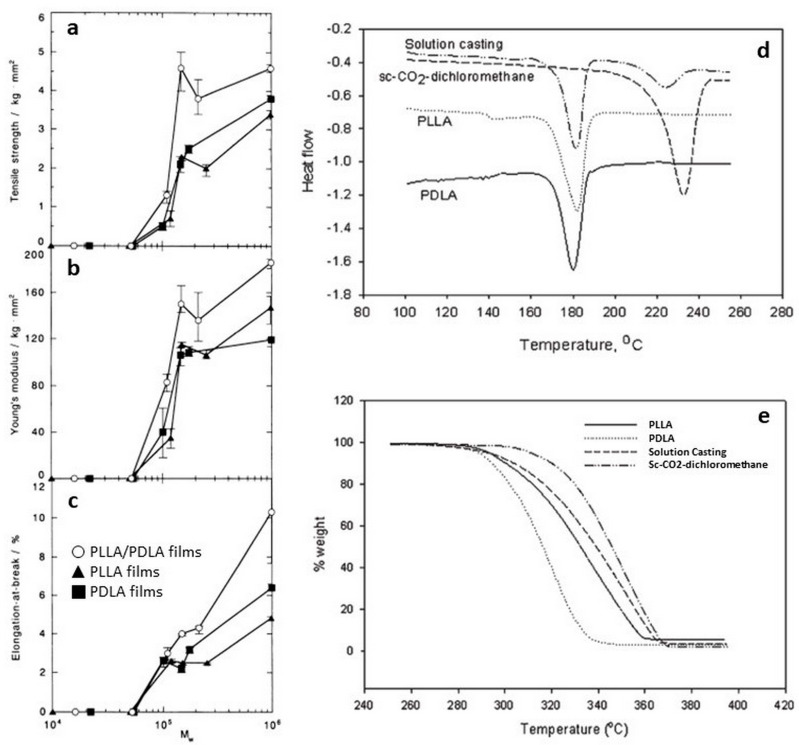
Properties of PLLA, PDLA and s-PLA (PLLA/PDLA) materials; mechanical properties as function of molecular weight. (**a**) Tensile strength (**b**) Young’s modulus (**c**) Elongation at break and thermal properties [14] (copyright and permission, Elsevier 2023). (**d**) Melting temperature. (**e**) Thermal degradation temperature [22] (Copyright and permission, American Chemical Society 2023). Reproduced from [22], with permission from American Chemical Society, 2023.

The mechanical, thermal, and physical properties of PLA homopolymers are affected by their molecular weight. The presence of s-PLA crystallites generated from PLLA/PDLA blends enhances the mechanical and thermal properties of the materials (Figure 2). As the properties of the PLA homopolymer are affected by its molecular weight, the mechanical properties of s-PLA from PLLA/PDLA blends are also a function of its molecular weight. The superiority of the mechanical properties of s-PLA is explained by the formation of a microphase structure upon 3D gelation, which suppresses the formation of large-scale spherulites [14]. The supercritical fluid method also has successfully produced perfect s-PLA materials with enhanced mechanical properties that are approximately 25% higher than those of the PLA homopolymers [22]. The melting temperature of s-PLA materials is approximately 230 °C (~50 °C higher than that of the PLA homopolymer), which is due to the strong bonds of the crystal network that hinder chain motion [8]. This strong interaction also disturbs and shifts thermal degradation to a higher temperature compared to that of the PLA homopolymer [6,22].

s-PLA can be obtained from a mixture of PLA homopolymers [2,3,4,5,14,16,19,20,21,22,26,27,28] copolymers, [9,28,29,30,35] and branched structures [23,36]. Structural modification affects the properties of synthesized s-PLA materials, including their crystallinity, morphology, stability, degradability, and mechanical properties [6,23,28,32,36,37,38]. Nevertheless, there are some constraints such as the molecular weights and stereocomplex memory (melt stability) that limit the development of s-PLA materials. Molecular weight is the main constraint on obtaining perfect s-PLA crystallites through solution-based and melt blending processes. The critical molecular weight of PLA homopolymers is *M_w_* ~1 × 10^5^ g/mol, which means a *M_w_* < 1 × 10^5^ g/mol lactide chain mobility will form 3D gelation through the stereocomplexation of PLA blends while the high-molecular-weight (*M_w_* > 1 × 10^5^ g/mol) PLA homopolymer dominates the crystallization of PLA blends [14]. The degree of crystallization of s-PLA results in substantial differences in thermal degradation (up to 25 °C of the onset of the thermal degradation point) and mechanical properties (tensile strength of 36.4 and 47.8 MPa for s-PLA with a ~25.9% crystallization degree) [22]. For industrial application, the suitable processing method is melt processing, which is fast, cheap, simple, does not require an organic solvent, and could be applied in most polymer production facilities. Linear s-PLA materials show drawbacks in stereocomplex memory (melt stability) during thermal processing. The s-PLA crystallization degree of linear PLLA/PDLA blends decreases after thermal processing [23,35,36]. For instance, the crystallization degree of a perfect s-PLA crystalline in a study decreased to 94.7% after hot pressing at 250 °C [22]. Biela reported that the high molecular weight of linear PLA blends (*M_n_* ~10,000 gr/mol) decreased their s-PLA degree after melting because the D- and L-lactide chains were dismantled when heated and could not be re-packed as s-PLA crystallites [36]. Recently, some researchers found that stereocomplex memory can be maintained by modifying the structure of PLLA or PDLA homopolymers by incorporating branched-structure [23,36] copolymerizing with flexible chains [35].

### 2.2. PLA Nanocomposites Synthesis and Properties

The present nanoscale fillers in the polymer matrix lead to substantial improvements in the mechanical, electrical, optical, thermal, and magnetic properties of the materials [33]. Based on their dimensions, there are four types of nanoscale fillers: zero-dimensional nanoparticles (particle size < 100 nm), one-dimensional nanofibers (diameter size < 100 nm), two-dimensional layered nanoplatelets (platelet’s thickness < 100 nm), and three-dimensional interpenetrating networks (3D size < 100 nm) [39]. Due to environmental concerns, bio-nanocomposites are materials of interest because they are a combination of natural or biodegradable polymers nanoscale organic or inorganic solid materials [40]. For PLA material development, the thermo-mechanical properties of PLA can be enhanced by incorporating nanosized fillers within the PLA matrix, known as PLA nanocomposites. Many researchers have reported various nanofillers of different sizes and shapes for the development of PLA nanocomposites [41,42,43,44,45,46,47,48,49,50,51,52,53,54,55,56,57,58,59,60,61,62,63,64,65]. The addition of nanofillers to PLA nanocomposites generally focuses on the improvement of the physical properties of PLA. For instance, clays are used to improve the mechanical and barrier properties [41,42,43,44,45,46,47], graphene and CNT are used to improve mechanical, barrier and electrical conductivity properties [48,49,50,51,52], chitosan is used in food packaging materials to improve food shelf life [53,54,55], tricalcium phosphate and hydroxyapatite are used to improve cytocompatibility [56,57,58,59,60,61], and silver and zinc oxide nanoparticles are used to confer anti-bacterial properties [62,63,64,65].

**Figure 3:**
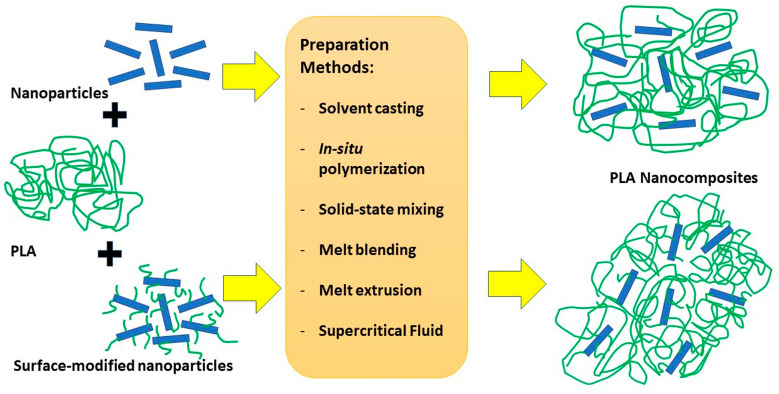
Preparation methods of PLA Nanocomposites.

For more than 20 years, numerous methods have been developed for the synthesis of PLA nanocomposites (Figure 3) [39]. Generally, PLA nanocomposites were prepared using organic solvents [57,59,62,63], melting/thermal processing [42,43,44,45,46,47,48,49,50,51,52,53,54,55,60,64,65], and supercritical fluid technology [66,67]. Solvent casting is a simple method to prepare PLA nanocomposites by mixing PLA homopolymers and nanoparticles, but evaporating the residual solvent takes a long time, so this method is not suitable for large-scale production. In solvent casting, nanoparticle surface modification, molecular weight and concentration are critical points in obtaining a homogeneous blend of PLA and nanoparticles [57]. In situ polymerization supports PLA–nanoparticle interaction because the polymer chains are grafted onto the nanoparticle surface, but this method shows limitations for obtaining high-molecular-weight grafted polymer chains [67]. Solid-state, melt blending and melt extrusion methods are common in the polymer industry because the material can be manufactured via the simple mixing of PLA and nanoparticles and controlling the temperature and processing time to minimize the potential loss via thermal degradation [39,42]. On the other hand, supercritical fluid can obtain homogeneous PLA nanocomposites but requires a high pressure and temperature, which results in a high production cost [66,67]. Among these methods, melt or thermal processing is the most suitable for the industry because it is compatible with existing processing facilities, and is also environmentally friendly because of the absence of organic solvents [39]. However, this method requires the control of nanoparticle dispersion to achieve enhanced properties and also the processing parameter (temperature and time) should be controlled for preventing the degradation of the PLA chains. The solution-based methods have limitations because evaporating the solvent takes a long time. Using supercritical fluid technology is a high-cost method to produce nanocomposite in batches.

Nanoparticles are added to PLA to improve the properties of the resulting nanocomposite materials. This enhancement of properties is related to the nanoparticle size, content, and dispersion in the polymeric matrix. Smaller particles (nanosized) are distributed more easily in the polymer matrix compared to micro-sized particles. Nanomaterial dispersion is the most critical parameter to achieve high-performance PLA nanocomposite materials [39]. To obtain adequate nanoparticle dispersions, material compatibility is crucial as it is related to polymer–filler interactions. The nanoparticle fillers in the PLA matrix act as nucleating agents to shorten the nucleation period and affect the nucleation transformation and crystallization rate up to a certain level [68,69]. The dispersion of nanoparticles in the PLA matrix can be characterized through transmission electron microscopy, tomography [70,71], small-angel X-ray scattering [72], and using a rheometer [73]. The dispersion of nanoparticles in the PLA homopolymer matrix is related to the interfacial bonding between the nanoparticles and the polymer matrix. Neat inorganic nanoparticles have weak interfacial bonding with the PLA matrix, which results in the formation of aggregates in PLA nanocomposites, affecting crystallization [74,75]. In PLA-based materials, nanoparticle dispersions can be improved by modifying the functionality of the nanoparticle surface and/or delaminating the layered nanoplatelets by applying organic modifiers [39,76]. Surface modification and PLA grafting are common strategies to improve the compatibility of nanoparticles in the PLA matrix [34,77,78,79,80]. There are many substances that can be utilized as a surface modifier, for instance, acyl chloride [34], acetic acid (for acetylation) [77,78], and organic acid (for esterification) [79,80]. Generally, surface functionality is modified to control the number of –OH functional groups as the starting point of the grafting of lactic acid or PLA chains on the surface of nanoparticles. A large number of –OH groups result in the high hydrophilicity of the nanoparticles, whereas a low number –OH groups results in grafted PLA chains with a high molecular weight [77,78]. For layered nanoplatelets such as clay, the exfoliated layer of the platelets can be obtained through ion-exchange chemistry to reduce van der Waals forces [39].

PLA nanocomposites are expected to possess better properties than those of neat PLA. The properties of materials depend on their structure and molecular interactions. PLA nanocomposites exhibit excellent thermal and mechanical properties if the blended materials are mixed homogenously, which is confirmed by the uniform dispersion of nanoparticles and their interfacial interactions. For instance, a small amount of MWCNT-g-PLLA contributed to the faster crystallization rate and higher glass transition temperature of a composite material [81]. The presence of 1 wt.-% of MWCNT-g-PLLA substantially increased the tensile modulus and tensile strength of PLA nanocomposites by up to 91% and 52%, respectively [82]. The addition of 3% graphene oxide-grafted PLLA resulted in a 37.8% tensile strength improvement compared to that of pristine graphene oxide in PLA composites [69]. The interfacial interactions among nanoparticles are presumably caused by hydrogen bonding between C=O groups of the PLA chains and the –OH functional groups on the surface of the nanoparticles [68,83]. The presence of nanoparticles may also result in specific or engineered properties, such as the antibacterial activity of silver and zinc oxide nanoparticles [63,64,65], and bone mineralization by hydroxyapatite [58,61,83] and nanodiamonds [84].

## 3. Stereocomplex Nanocomposite PLA (Stereo-Nano PLA)

PLA, as a “green” polymer, has sustainability and degradability characteristics; however, for some specific applications, PLA has limitations regarding its physical, thermal, and mechanical properties [1]. To solve this problem, stereocompexation [2,14,15,16,17,18,19,20,21,22,23,24,25] and nanocomposites [42,43,44,45,46,47,48,49,50,51,52,53,54,55,59,60,62,63,64,65,66,67] are suitable approaches. Considering their similarity in preparation methods, stereocomplexation and nanocomposite approaches may be combined to improve the thermal and mechanical properties of PLA-based materials [85,86]. This combination will offer more advanced and tunable properties for future material applications.

Stereo-nano PLA is an advanced class of biomaterials produced by combining PLLA/PDLA blends and nanoparticles through stereocomplexation and nanocomposite approaches. Many researchers have studied the development of stereo-nano PLA of several molecular weights containing various nanoparticles with outstanding improvements of their properties (Table 2).

The properties of stereo-nano PLA materials are affected by nanoparticle miscibility and the homopolymer’s molecular weight. High molecular weight is related to high physical, mechanical, and thermal properties but it makes it difficult to achieve a high degree of crystallinity for s-PLA. The combination of PLLA, PDLA, and nanoparticles can help to attain stereo-nano PLA materials with optimum properties in the molecular weight range of (*M_w_*) up to 253,000 g/mol [115].

Stereo-nano PLA can be produced mainly through solution-based and melt processing methods. The solution-based method is a simple approach and can be performed at a small scale in the laboratory. Meanwhile, melt processing is suitable for industry applications. In solution-based methods, the solubility of the PLA homopolymers and nanoparticles in the solvent is crucial for controlling the miscibility and dispersibility of the materials. Chloroform, dichloromethane, and tetrahydrofuran are suitable solvents for PLA homopolymers because they are close to the solubility parameters of PLA [22,133]. The solubility of the PLA homopolymer in a solvent also depends on its molecular weight and concentration. A PLA matrix of a low molecular weight and a low concentration will dissolve easily in the selected organic solvent (chloroform, dichloromethane, and tetrahydrofuran) with low viscosity. The low-viscosity solution allows the mobility of the free chains of L-lactide and D-lactide to initiate the formation of a s-PLA crystallite through hydrogen bonding between CH_3_–O=C of PDLA and PLLA chains [6]. In melt processing, the temperature affects the melt viscosity of the PLA blends. A higher temperature results in a lower melt viscosity, but also affects the chain degradation of PLA homopolymers.

**Figure 4:**
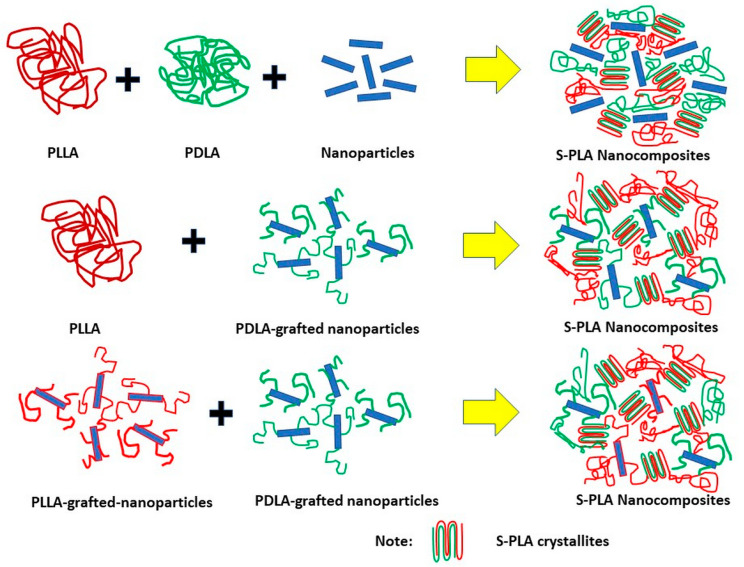
Material combination of stereo-nano PLA.

In the preparation of stereo-nano PLA, the types of nanoparticles also influence their solubility and dispersibility in the PLLA/PDLA blends. There are several material combinations that can be used to produce stereo-nano-PLA (Figure 4):

(i) PLLA + PDLA + nanoparticles
  The first and most simple combination to form stereo-nano PLA comprises PDLA, PLLA, and nanoparticles. The chemical structure of the nanoparticle is critical to ensure its miscibility in the PLLA/PDLA matrix. The –OH and C=O groups in lignin enhance the mixing capability with PLLA/PDLA blends through hydrogen bonding [93]. Despite MWCNT in PLLA/PDLA blends being able to yield stereocomplex crystallites, the poor dispersion of MWCNT in the polymer matrix leads to agglomerates and limits stereocomplex crystallization [95]. The presence of organic modifiers in nanoplatelets helps their delamination and improves their dispersion in the PLLA/PDLA matrix [86,101,102]. The presence of 1–10% PDLA in the PLLA matrix prevents the aggregation of carbon black (CB) and helps the formation of a CB nanoparticle network [110,117]. Multifunctional CB increases its compatibility with the PLLA/PDLA matrix [118]. The presence of –OH groups of CNF results in well-dispersed CNF in the PLLA/PDLA matrix [115]. An increasing nanosilica content results in the formation of aggregates, although it also aids in the formation of the stereocomplex crystallites in PLLA/PDLA blends [126]. The functionalization of nanoparticles affects their compatibility and dispersion in the PLLA/PDLA matrix and also affects the formation of stereocomplex crystallites.
(ii) PLLA + PDLA-grafted nanoparticles
  A high content of non-functionalized nanoparticles leads to aggregation in the polymer matrix. The functionalization of nanoparticles enhances the miscibility and interfacial interaction between the nanoparticle surface and the PLA chains. A combination of PLLA/nanoparticles-g-PDLA shows better crystallization compared to PLLA/PDLA blends. This is explained by the reduction in the crystallization activation energy by nanomaterials, such as CNT and graphene oxide [88,89]. The PDLA chains grafted on the CNT particles increase the stability of the nanoparticles in the PLLA matrix by forming stable stereocomplex crystallites through interfacial adhesion [90,107,111,116,128].
(iii) PLLA-grafted nanoparticles + PDLA-grafted nanoparticles

The combination of PLLA/nanoparticles-g-PDLA results in substantial improvements in s-PLA formation. Moreover, nanoparticles-g-PLLA/nanoparticles-g-PDLA exhibits enhanced s-PLA crystallite formation [23,99,125]. The combination graphene-g-PLLA/graphene-g-PDLA improves interfacial interaction to form s-PLA crystallites, whereas graphene nanoparticles act as heterogeneous nucleation sites [125].

The combination of PLLA- and PDLA-grafted nanoparticles shows advantages in the faster formation of s-PLA crystallites and boosts the stereocomplex memory during thermal processing. The properties of stereo-nano PLA are enhanced by s-PLA crystallites and the dispersion of nanoparticles in the polymeric matrix.

## 4. Properties and Applications of Stereo-Nano PLA

Tunability is one of the main concerns in material development because some applications require materials with specific characteristics. In packaging applications, anti-bacterial activity is important for preventing food degradation due to bacterial contamination. Controlling mechanical strength is essential in bone fixation/regeneration, but other properties such as biocompatibility, bioactivity, and osteoconductivity are also required. Photothermal therapy in cancer treatment requires materials capable of absorbing near-infrared (NIR) light and producing localized heat during NIR irradiation. To achieve these specific properties, the combination of materials is mandatory. Stereo-nano PLA offers this possibility as organic and inorganic materials can be combined to achieve specific properties.

**Figure 5:**
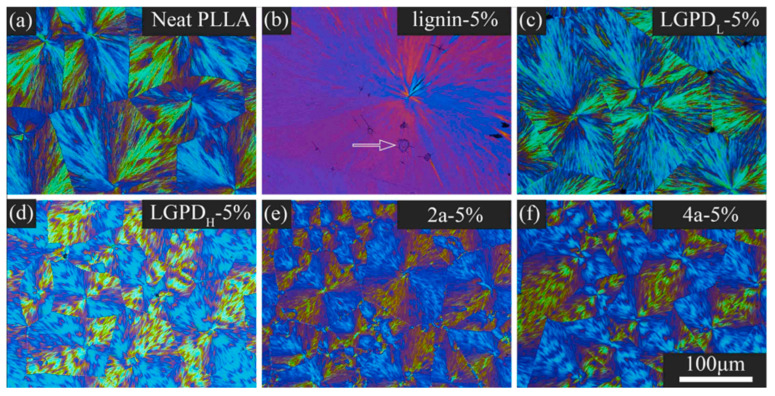
Polarized optical microscope images of spherulites of neat PLLA and various PLLA blends after isothermal crystallization at 135 °C for 6 h: (**a**) neat PLLA, (**b**) 5% lignin (PLLA with 5% lignin content), (**c**) 5% LGDPD_L_ (PLLA with 5% lignin-grafted PDLA; 1100 Da block length), (**d**) 5% LGDPD_H_ (PLLA with 5% lignin-grafted PDLA; 2000 Da block length), (**e**) 5% 2a (PLLA with 5% 2-armed PDLA), (**f**) 5% 4a (PLLA with 5% 4-armed PDLA). Reproduced from [130], with permission from Elsevier, 2022.

The combination of stereocomplexation and nanocomposite approaches offers simultaneous enhancements of the physical, mechanical, thermal, and other properties of stereo-nano PLA materials. The properties of stereo-nano PLA depend on the material combination and nanoparticle functionality. Chain mobility determines the formation of s-PLA crystallites in stereo-nano PLA. Blends of linear PLLA and PDLA are restricted by their molecular weight [14], while nanoparticles act as hetero-nucleating agents. PLLA and PDLA covalently bonded onto nanoparticles surfaces offer special benefits for obtaining s-PLA crystallites because of the limited mobility of the grafted PLLA and PDLA chains. Specific properties can be conferred to stereo-nano PLA depending on the type of nanoparticles used. Some nanoparticles work as nucleating agents to improve the crystallinity of PLA, in turn enhancing its mechanical and thermal properties [89,90,91,92,94,99,100,101,102,126,127,130]. PDLA grafted onto lignin supports the formation of s-PLA crystallites, improves lignin dispersion, accelerates the crystallization rate, and results in the greater hydrolysis resistance of PLLA/PDLA blends [130]. As shown in Figure 5, PDLA grafted onto lignin affects the crystallization rate by increasing the dispersibility of lignin particles in the matrix and the formation of s-PLA crystallites as confirmed by the smaller spherulite size compared to that of neat PLLA.

**Figure 6:**
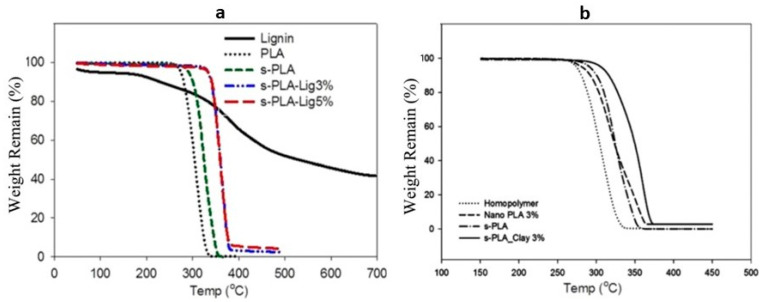
(**a**) Thermal degradation profile of lignin, PLA homoplymer, s-PLA and stereo-nano-PLA (3% s-PLA-Lig and 5% s-PLA-Lig) [98]. (**b**) Thermal degradation profile of PLA homopolymer, PLA nanocomposite with 3% of clays (3% nano PLA), s-PLA, and stereo-nano PLA with 3% of clays (3% s-PLA_Clay). Reproduced from [85], with permission from Springer Nature, 2022.

The presence of nanoparticles in stereo-nano PLA also substantially improves the thermal degradation properties (Figure 6). Biodegradable blends of s-PLA and lignin exhibit doubled thermal degradation improvement compared to neat s-PLA as a consequence of the char formation of lignin and the s-PLA crystalline structure [96]. The intercalated structure of clay in stereo-nano PLA materials also results in the synergetic improvement of thermal degradation in comparison with that in s-PLA and PLA nanocomposites as a result of the strong interaction between the L- and D-lactide chains, which provide a thermal insulator in addition to the mass transport barrier provided by the clay particles [85,86]. In crystallization studies, the presence of graphene oxide lowers the crystallization activation energy of s-PLA crystallites, reduces chain mobility and hinders crystal growth as a consequence of the exfoliation of graphene oxide sheets [89]. The presence of graphene oxide nanoplatelets accelerates the crystallization time by 2.7 min compared to the half-time crystallization of s-PLA [92]. The D-lactide chains on graphene nanoplatelets promote the formation of s-PLA crystallites at the interface of the PLLA matrix and exhibit a synchronous enhancement in thermal conductivity, heat distortion temperature, and mechanical properties [125]. The simultaneous improvement in thermal and mechanical properties represents an opportunity for the applications of PLLA-based materials in high-performance materials and next-generation microelectronic devices.

**Figure 7:**
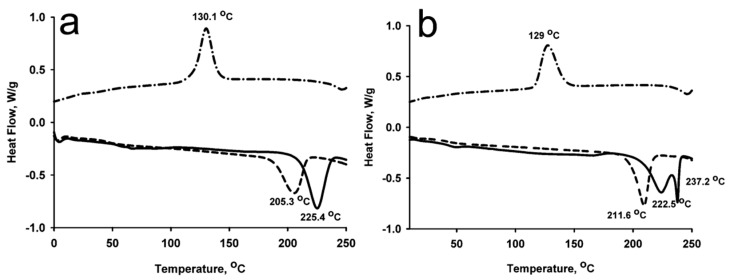
The DSC thermograms of stereo-nano PLA from MWCNT-g-PLLA and MWCNT-g-PDLA. (**a**) Precipitated s-PLA-21 and (**b**) thin-film s-PLA-21. The sample was heated from 0 to 250 °C, cooled down to 0 °C, kept for 1 min, and heated to 250 °C with a heating and cooling rate of 10 °C; the solid line represents the first heating run, and the dashed line represents the second heating run. Reproduced from [93], with permission from American Chemical Society, 2023.

Adding a carbon-based nanofiller into stereo-nano PLA materials results in many property improvements [90,93,95,104,105,110,117,118]. The presence of a small amount of MWCNT in low-molecular-weight PLLA/PDLA blends enhances stereocomplex crystallization [95]. PLA-functionalized MWCNTs boost the formation of stereocomplex crystallites. The stereo-nano PLA from equimolar MWCNT-g-PLLA and MWCNT-g-PDLA blends exhibits unusual thermal properties such as a reversible stereocomplex memory (melt stability) (Figure 7) [93]. A small content (4%) of PDLA-functionalized CNWs can improve the tensile strength up to 45.3% but also decreases the elongation at break [104]. The stereo-nano PLA generated from PLLA/PDLA and CB exhibits a unique self-networking capability; the high PDLA and CB content prevents CB aggregation by forming s-PLA crystallites and facilitating the networking of the CB nanoparticles [110,117,118]. The electrical characteristics of MWCNTs and CB in stereo-nano PLA also improve the electrical conductivity [105]. The 3D structure of CNT aids in the formation of 3D continuous networks for scaffold materials [90]. The improvements in stereocomplex memory, thermal, mechanical, and electrical properties as a result of the incorporation of CNT and CB nanoparticles represent potential applications for stereo-nano PLA in engineering, electronic, high-performance and scaffold materials.

As a plant-based material, cellulose (cellulose nano whiskers/nanofibers/nanocrystals) is suitable as a bio-based nanofiller in stereo-nano PLA materials. The –OH functional groups of cellulose can be utilized as a grafting co-initiator for D- or L-lactide polymerization [94,99,103,106,107]. Stereo-nano PLA containing PLA-grafted cellulose exhibits an excellent crystallization rate and stereocomplex memory [99,103]. Blends of PLLA-g-CNW and PDLA-g-CNW show unusual behavior at low and high concentration. At a low concentration, these blends easily form a cast film with a high degree of s-PLA crystallites, whereas, at a high concentration (20% *w*/*v*), they directly form a gel containing s-PLA crystallites [99]. The cellulose nanofillers in stereo-nano PLA also enhances the s-PLA crystalline structure and dispersion of the nanofillers, resulting in improved thermal and mechanical properties [94,99,103], heat distortion resistance [106], and oxygen and water vapor permeabilities [107], which align with the requirement for high-temperature engineering and packaging applications.

**Scheme 1:**
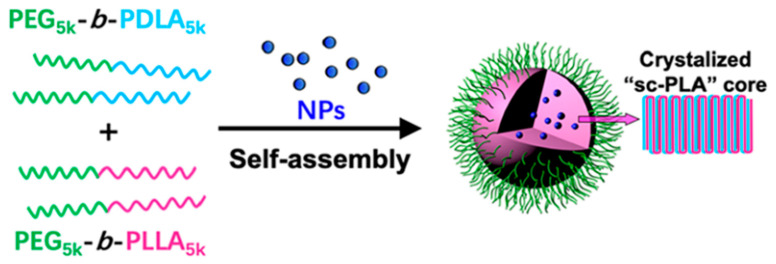
Schematic illustration of stereo-nano PLA micelles with encapsulating stability supported by stereocomplex formation. Reproduced from [120], with permission from American Chemical Society, 2023.

Stereo-nano PLA has also been applied in bone tissue engineering. For this purpose, organic–inorganic blends of PLA and hydroxyapatite are crucial. PDLA grafted on hydroxyapatite substantially improves the dispersion of the inorganic nanofiller in the polymer matrix and stereocomplexation strengthens the mechanical properties [111,121,128]. A study reported a stereo-nano PLA containing s-PLA and hydroxyapatite that exhibited enhanced type I collagen expression and formation of bone-like nodules [121] as well as high cytocompatibility [128]. Magnesium hydroxide nanoparticles with anti-inflammatory and antibacterial effects provide advantages to stereo-nano PLA materials in biomedical applications [129]. Silver nanowire in stereo-nano PLA boosts thermal conductivity, which is advantageous for thermal management materials and microelectronic devices [119]. The interactions between L- and D-lactide chains drive the self-assembly of micelles in aqueous solutions (Scheme 1) [120]. Molecular stereocomplexation enhances the stability of encapsulated ferrite-based nanoparticles, which could be used as magnetic resonance contrast agents in cancer diagnosis applications [120]. Stereocomplex micelles could also be used as nanocarriers for gold nanoparticles in photothermal cancer treatment and chemotherapy [123].

## 5. Prospective and Conclusions

This review collected and discussed research on the development of stereo-nano PLA as an advanced material. Stereo-nano PLA can be generated by combining stereocomplexation and nanocomposite methods because both approaches are similar. The studies mentioned in this review describe the step-by-step development of stereo-nano PLA to overcome PLA’s limitations. The stereocompexation of PLA homopolymer blends results in the formation of new crystallite structures associated with D- and L-lactide hydrogen bonding interactions. In addition, nanocomposites exhibit enhanced thermal and mechanical properties as a result of the incorporation of a nanofiller in the polymer matrix, followed by nucleation transformation that accelerates the crystallization rate of the materials. In stereo-nano PLA, D- and L-lactide fragments interact, aiding in the formation of s-PLA crystallites, while nanoparticles act as hetero-nucleating agents, which results in synergism and superior material properties. Modification of the molecular structure of the PLA homopolymers also alters the thermal and mechanical properties of stereo-nano PLA, including its capability to maintain stereocomplex memory, which is critical for industrial processing. The type of nanoparticles and the interfacial interaction between the nanoparticles surface and the polymer matrix are crucial for determining the final properties of stereo-nano PLA materials. The interfacial interaction correlates with the dispersion of the nanoparticles in the polymeric matrix and can be controlled by surface modification or polymer grafting. Special features of the nanoparticles may contribute additional characteristic to stereo-nano PLA. These beneficial properties represent an opportunity for stereo-nano PLA materials as future advanced materials.

The molecular weight constraint on s-PLA formation can be diminished by incorporating PLA on the surface of nanoparticles, modifying the multi-branch structure of the PLA homopolymers, and applying copolymerization strategies. The superior properties of stereo-nano PLA represent a great opportunity to replace fossil-based polymers in engineering applications. Specific characteristics, such as anti-inflammatory, antibacterial, photothermal, and electrical conductivity properties are conferred by nanomaterials and are advantageous for biomedical, electronic, diagnostic, and packaging applications. High-molecular-weight PLA is not the only material that can be used for synthesizing stereo-nano PLA. Low-molecular-weight PLA-based mixtures with sterocomplex self-assembly capability can be utilized as micelle nanocarriers. These micelles may be developed for specific therapeutics and drug delivery systems by controlling the molecular weight and structure and incorporating specific nanoparticles. Therefore, combining s-PLA crystallites and nanoparticles is advantageous as it results in stereo-nano PLA materials that could be used as advanced materials with biodegradable and tunable properties.

## Figures and Tables

**Table 1:** Synthesis of s-PLA materials and its development findings.

Polymers	Processing	Condition	s-PLA Shape	Findings	Ref.
PLLA and PDLA (*M*_V_ = 7.0 × 10^4^ g/mol)	Solution-precipitation	Solvent: dichloromethane-methanol Variable: PLLA:PDLA ratio Concentration: 1.0 g/dL	Fibrous materials	Formation of s-PLA crystallites was observed via a differential scanning calorimeter (DSC) and X-ray diffraction (XRD) analysis	[2]
PLLA (*M*_W_ = 150,000 g/mol) and PDLA (*M*_W_ = 100,000 g/mol)	Solution casting	Solvent: chloroform Fixed PLLA:PDLA ratio (1:1) Concentration: 1.0 g/dL	Films	Formation of s-PLA was caused by CH3–O=C interaction based on FT-IR	[4]
PLLA and PDLA	Solution casting	Solvent: dichloromethane Variable: molecular weight Fixed PLLA:PDLA ratio (1:1) Concentration: 1.0 g/dL	Films	In the range *M*_W_ 1 × 10^5^–1 × 10^6^, PLLA/PDLA blends show higher mechanical properties caused by s-PLA crystallites	[14]
PLLA and PDLA (*M*_V_ = ~4.0 × 10^4^ g/mol)	Sol-Gel process	Solvent: chloroform Variable: PLLA:PDLA ratio Concentration: 7.5–17.5 g/dL	Gels, microgels	Rapid complex formation at 1:1 ratio at higher concentrations	[15]
PLLA and PDLA (*M*_V_ = ~1.0–9.9 × 10^4^ g/mol)	Solution casting	Solvent: dichloromethane Variable: optical purity Fixed PLLA:PDLA ratio (1:1) Concentration: 1.0 g/dL	Films	High optical purity affects the formation of s-PLA crystallites more than their homocrystallites	[16]
PLLA (*M*_V_ = 1.32 × 10^4^ g/mol) PDLA (*M*_V_ = 4.42 and 1.45 × 10^4^ g/mol)	Melt crystallization	Variable: optical-purity PDLA, 80% and 100% Concentration:2.0 g/dL	Films	High optical purity supports the fast rate of the s-PLA formation	[17]
PLLA and PDLA	Melt crystallization	Variable: Molecular weight, PLLA/PDLA ratio Nucleation comparison	Films	The s-PLA crystallites were form at low concentration of PDLA. The s-PLA nucleation effect was superior to that of talc and PLLA homopolymers.	[18]
PLLA and PDLA	Melt crystallization	Variable: molecular weight, PLLA/PDLA ratio, crystallization temperature and time	Films	The addition of PDLA to PLLA formed s-PLA crystallites which acted as nucleation site and improved its properties	[19,20,21]
PLLA and PDLA	Supercritical CO_2_-cosolvent	High-molecular-weight, fixed 1:1 ratio Variable: solvent type, pressure, temperature, and time	Dry powder	Rapid formation of 100% s-PLA from high-molecular-weight PLA in dry powder shapes	[22,23]
PLLA and PDLA	Supercritical fluid	Variable: molecular weight, Fixed 1:1 ratio, pressure, temperature, and time	Dry powder	Supercritical fluid parameter affects the degree of s-PLA formation and final s-PLA shapes	[24,25]

Note: *M*_V_: viscosity average molecular weight; *M*_W_: weight average molecular weight.

**Table 2:** Development of stereo-nano PLA.

PLA Matrix	Nanoparticles/Modification	Preparation Methods	Property Improvement	Ref.
PLLA and PDLA (*M*_n_ = 25,000–75,000 g/mol)	Cloisite 30 B Fluorinated clay	Supercritical fluid	Increased melting temperature (up to 64 °C) and thermal degradation temperature (up to 30 °C)	[85,86]
Star-shaped PLLA and PDLA (*M*_W_ = 12,000–35,000 g/mol)	Octafunctional polyoctahedral silsesquioxanes (POSS) as initiator (core of star polymer)	Solution method by tetrahydrofuran (THF)	Self-formation of s-PLA, increased melting temperature, doubled improvement of hardness (GPa)	[87]
Commercial PLLA (*M*_n_ = 130,000 g/mol)	CNT-grafted PDLA Graphene oxide-grafted PDLA	Solution casting in chloroform	Crystallinity and mechanical properties	[88,89,90,91,92]
MWCNT-PLLA MWCNT-PDLA	Multiwall CNT (MWCNT) as initiator (core of grafted PLA)	Solution casting in chloroform	Reversible s-PLA crystallization after melting (stereocomplex memory)	[93]
Commercial PLLA (*M*_n_ = 130,000 g/mol)	Cellulose nanocrystal -grafted PDLA	Melt blending in mini-extruder	Thermal and mechanical properties	[94]
PDLA; PLLA; pyrene-end-functionalized PLLA	Pristine MWCNT	Solution casting in chloroform	Pyrene end-group prevention of the formation of crystalline s-PLA, and enhancement of w-PLA crystallization by MWCNT but with poor dispersion	[95]
PDLA (*M*_n_ = 126,537 g/mol) PLLA (*M*_n_ = 194,597 g/mol)	Lignin	Supercritical carbon dioxide—solvent	Thermal degradation properties	[96]
PLLA and PDLA block copolymer	Methacrylisobutyl POSS (MA-POSS)	Solution casting in THF	Self-assembly stereocomplex, thermal properties	[97]
PLLA	Magnesium oxide–oligo D-lactide (MgO-ODLA)	Solution casting in chloroform	Enhanced mechanical properties and suppressed severe acid-induced inflammation.	[98]
PLA-graft-cellulose nanowhiskers (PLLA-g-CNW; PDLA-g-CNW)	Acetylated-CNW	Solution casting in chloroform	Crystallization, thermal and mechanical properties, stereocomplex memory	[99]
PLLA (*M*_n_ = 100,000 g/mol) and Cloisite 30B-g-PDLA	Cloisite 30 B	Melt process	Thermal and mechanical properties	[100]
PLLA (*M*_n_ = 132,000 g/mol) PDLA (*M*_n_ = 64,800 g/mol)	Oleylamine zinc phenylphosphonate	Solution casting in dichloromethane	Enhanced thermal and mechanical properties, higher disintegration rate compares neat s-PLA	[101,102]
PLLA (*M*_n_ = 51,000 g/mol) PDLA (*M*_n_ = 86,000 g/mol)	Cellulose nanocrystalline (CNC) grafted PLLA	Solution casting in chloroform, evaluated after melting	CNC-g-PLLA/PDLA with a higher s-PLA degree after cooling, and higher crystallinity	[103]
PLLA (*M*_W_ = 200,000 g/mol)	Functionalized CNT (Branched PDLA-g-CNT)	Melt blending in mini extruder	Excellent thermal stability, and higher tensile strength (62.5 MPa)	[104]
PLLA (*M*_W_ = 170,000 g/mol) PDLA grafted ethylene-acrylic ester (*M*_W_ = 96,000 g/mol)	MWCNT	Melt mixing at 190 °C	Improve interfacial strength and impact toughness)	[105]
PLLA (*M*_W_ = 100,000 g/mol)	Cellulose nanocrystalline (CNC) grafted PDLA	Solution casting in chloroform	Enhanced crystallization, storage modulus, and heat distortion temperature	[106]
PLLA (*M*_W_ = 239,000 g/mol) PDLA (*M*_W_ = 182,000 g/mol)	PDLA-grafted-cellulose microcrystals (CMC)	Melt process using twin-screw extruder	Enhanced gas barrier and thermomechanical properties	[107]
Commercial PLLA	8-arms POSS-(PDMAEMA-b-PDLA)_8_	Solution casting in chloroform	Enhanced thermal and mechanical properties up to certain content nanoparticle, higher disintegration rate compares neat s-PLA	[108]
PLLA	SiO_2_-r-PDLA nanoparticles	Solution blending, injection molding	Improved interface control, thermal and mechanical properties	[109]
PLLA (*M*_W_ = 170,000 g/mol) PDLA (*M*_W_ = 167,000 g/mol)	Carbon black	Melt mixing	Enhanced matrix crystallization and mechanical properties	[110]
PLLA (*M*_W_ = 100,000 g/mol)	PDLA-grafted-nanohydroxyapatite (nanoHA)	Solution casting in chloroform	Enhanced interfacial adhesion, crystallization, mechanical and thermal properties	[111]
PLLA (*M*_n_ = 180,000 g/mol) PDLA (*M*_n_ = 70,000 g/mol)	PLLA-grafted-ZnO	Solution casting in chloroform	Improved crystallization rate, non-leaching properties	[112]
PLLA (*M*_n_ = 143,000 g/mol)	PDLA-b-PDMA-Starch nanoparticles	Solution casting in chloroform	Synergetic effect on thermal and mechanical properties	[113]
PLLA and PDLA (*M*_W_ = 100,000 g/mol)	Carbon quantum dots (CQD)	Solution electrospinning	High shear piezoelectricity, photoluminescence and improved heat resistant	[114]
PLLA (*M*_W_ = 253,000 g/mol) PDLA (*M*_W_ = 100,000 g/mol)	CNCs	Solution casting in dichloromethane	Improve hydrolytic degradation rate and heat resistant	[115]
PLLA (*M*_n_ = 180,000 g/mol)	TiO_2_@SiO_2_-g-PDLA	Solution casting in chloroform	Enhanced mechanical properties (by 49%), UV shielding and UV resistance	[116]
PLLA (*M*_W_ > 160,000 g/mol) PDLA (*M*_W_ > 185,000 g/mol)	Carbon black, multifunctional carbon black	Melt mixing	Crystallinity, electrical properties	[117,118]
PLLA (*M*_W_ = 210,000 g/mol) PDLA (*M*_W_ = 200,000 g/mol)	Silver nanowires (AgNWs)	Electrospinning–dip coating	Thermal conductivity	[119]
PEG_5k_-b-PDLA_5k_ PEG_5k_-b-PLLA_5k_ (PEG: polyethyleneglycol)	MnFe_2_O_4_ MnFe_2_O_4_@Fe_3_O_4_	Solution dispersion	Biocompatibility and superparamagnetic properties Excellent negative contrast enhancement of MR signals	[120]
PLLA (*M*_W_ = 210,000 g/mol)	HA-g-PDLA	Electrospinning	Mechanical properties, BMSC proliferation, osteogenic differentiation.	[121]
PLLA (*M*_n_ = 150,000 g/mol)	CNCs-PLLA CNCs-PDLA	Solution casting in dichloromethane	Crystallinity (up to 86.7%), mechanical properties (up to 36%)	[122]
Poly(2-(dimethylamino) ethyl methacrylate)-block-poly(D-lactide) (PDMAEMA-PDLA) PEG-PLLA	Gold nanoparticles	Solution mixing	The light-responsive drug release and therapeutic efficacy	[123]
PLLA, PDLA	Cellulose nanofibers (CNF) PLLA-g-graphene PDLA-g-graphene	Solution precipitation followed by compression molding	Heat resistance and thermal conductivity	[124,125]
PLLA (*M*_W_ = 207,000 g/mol) PDLA (*M*_W_ = 110,000 g/mol)	Nanosilica (AEROSIL 200)	Melt blending	Thermal stability (~33 °C higher), storage modulus, tensile strength and modulus	[126]
PLLA (*M*_W_ = 74,000 g/mol)	SiO_2_-PDLA PLLA-g-GMA (compatibilizer)	Melt mixing–3D printing	Heat resistance and tensile strength	[127]
PLLA (*M*_W_ = 88,400 g/mol)	PDLA-g-nanoHA	Selective laser sintering (SLS)	Interfacial bonding, cytocompatibility	[128]
PLLA	Mg(OH)_2_-g-Oligo(D-lactide-co-caprolactone) (MH-ODLCL) S-PLA microparticles	Solution casting–compression molding	Mechanical properties, anti-inflammatory	[129]
PLLA	Lignin-g-PDLA Lignin-g-2-armed PDLA Lignin-g-4-armed PDLA	Solution casting in Chloroform	Thermal and mechanical properties	[130]
PLLA (*M*_W_ = 170,000 g/mol) PDLA (*M*_W_ = 130,000 g/mol)	Cellulose nanofibers	Melt compounding	Interchain molecular interactions, crystallization rate	[131]
PLLA (*M*_n_ = 68,000 g/mol) Poly(1,4-butylene succinate (PBSU) (*M*_n_ = 68,000 g/mol)	PDLA-PBSU-g-nanorod (AlOOH-g-(D&B) PLLA-PBSU-g-nanorod (AlOOH-g-(L&B)	Melt blending	Interfacial stabilization, phase compatibility, thermal resistance, high modulus, fast crystallization rate	[132]

Note: *M*_n_: number average molecular weight; *M*_W_: weight average molecular weight.

## Data Availability

Data are contained within the article.
